# MDM2/X Inhibitors as Radiosensitizers for Glioblastoma Targeted Therapy

**DOI:** 10.3389/fonc.2021.703442

**Published:** 2021-07-08

**Authors:** Xanthene Miles, Charlot Vandevoorde, Alistair Hunter, Julie Bolcaen

**Affiliations:** ^1^ Radiobiology, Radiation Biophysics Division, Nuclear Medicine Department, iThemba LABS, Cape Town, South Africa; ^2^ Radiobiology Section, Division of Radiation Oncology, Department of Radiation Medicine, University of Cape Town and Groote Schuur Hospital, Cape Town, South Africa

**Keywords:** p53, MDM2 & MDMX, radiation, glioblastoma, radiosensitizer, radiotherapy, targeted therapy

## Abstract

Inhibition of the MDM2/X-p53 interaction is recognized as a potential anti-cancer strategy, including the treatment of glioblastoma (GB). In response to cellular stressors, such as DNA damage, the tumor suppression protein p53 is activated and responds by mediating cellular damage through DNA repair, cell cycle arrest and apoptosis. Hence, p53 activation plays a central role in cell survival and the effectiveness of cancer therapies. Alterations and reduced activity of p53 occur in 25-30% of primary GB tumors, but this number increases drastically to 60-70% in secondary GB. As a result, reactivating p53 is suggested as a treatment strategy, either by using targeted molecules to convert the mutant p53 back to its wild type form or by using MDM2 and MDMX (also known as MDM4) inhibitors. MDM2 down regulates p53 activity *via* ubiquitin-dependent degradation and is amplified or overexpressed in 14% of GB cases. Thus, suppression of MDM2 offers an opportunity for urgently needed new therapeutic interventions for GB. Numerous small molecule MDM2 inhibitors are currently undergoing clinical evaluation, either as monotherapy or in combination with chemotherapy and/or other targeted agents. In addition, considering the major role of both p53 and MDM2 in the downstream signaling response to radiation-induced DNA damage, the combination of MDM2 inhibitors with radiation may offer a valuable therapeutic radiosensitizing approach for GB therapy. This review covers the role of MDM2/X in cancer and more specifically in GB, followed by the rationale for the potential radiosensitizing effect of MDM2 inhibition. Finally, the current status of MDM2/X inhibition and p53 activation for the treatment of GB is given.

## Introduction

The classification of gliomas is traditionally based on histologic type and malignancy grade. It varies from low grade glioma, classified as benign with a high curative chance, to high grade glioma which is typically associated with rapid proliferation linked to disease evolution (grade I - IV). Since 2016, the World Health Organization (WHO) classification no longer relies solely on histological criteria but incorporated additional molecular biomarkers to improve diagnosis and prognosis of glioma patients. Especially the use of molecular techniques, such as arrays and next generation sequencing, play an integral role in the identification of mutations in gliomas (1, 2). Glioblastoma multiforme (GB) is classified as a grade IV, the highest grade in the WHO classification of brain tumors, and is the most common malignant central nervous system (CNS) tumor with a global incidence of 0.59–3.69 per 100 000 (3–5).

Despite numerous attempts over the past decade to find more effective treatments, the standard care for GB has remained essentially unchanged. This involves maximal safe surgical resection, external beam radiation therapy (EBRT) plus concomitant and adjuvant chemotherapy using the alkylating agent temozolomide (TMZ) - this is known as the Stupp protocol (6). Various avenues have been explored to improve GB therapy, such as targeting the immune system through gene therapy, viral vectors and targeted drug therapy to name a few (7, 8). Sadly, despite multiple clinical trials, median survival from diagnosis is still only 15-17 months (1, 6, 9–12). Treatment challenges often derive from the molecular and cellular heterogeneity inherent to these tumors. They include innate and acquired resistance with subpopulations of tumor cells harboring stem-like properties rendering them more resistant to therapy (13–15). Another major challenge in GB patients is tumor recurrence, which is unfortunately inevitable and results in a more aggressive and radioresistant secondary tumor. The standard of care for patients with recurrent GB is not well defined (1).

There has been an increased interest in the molecular pathogenesis of malignant tumors and this led to the development of monoclonal antibodies (mAbs) and small molecule (SM) inhibitors blocking critical pathways involved in tumor resistance and progression. These include the targeting of DNA repair pathways, cell cycle control enzymes/genes and their downstream pathways, as well as growth factor receptors (16, 17). Secondly, these targeted drugs can often function as radiosensitizers to enhance the cytotoxicity of subsequently administered radiation therapy (RT) while minimizing deleterious side effects towards surrounding normal tissues (18, 19).

In this review, the rationale for influencing the p53 and mouse double minute 2 (MDM2) pathway as a radiosensitizing and therapeutic strategy for GB will be covered. 84% of GB patients show a deregulation of the p53-MDM2 pathway (4, 20). MDM2 plays an imperative role in down regulating p53 activity *via* ubiquitin-dependent degradation and is amplified or overexpressed in 14% of GB cases. Hence, suppression of MDM2 through different approaches, offers an opportunity for urgently needed new therapeutic interventions for GB. In addition, the combination of MDM2 inhibitors with ionizing radiation (IR) may offer a valuable therapeutic radiosensitizing strategy by influencing the DNA damage response (21). Since the release of the structure of the MDM2–p53 interaction 25 years ago (22), numerous SM MDM2 inhibitors have been discovered and investigated, including SAR405838, HDM-201, NVP-CGM097, MK-8242, RG7112, RG7388, ALRN-6924 and AMG232 (23–31). Many of these inhibitors are currently being investigated in clinical trials as novel cancer treatments. The growing interest is reflected by the amount of reviews published in the last years (30–41). However, to date, only a limited number of MDM2 inhibitors have been tested for the treatment of GB or in combination with RT.

## Radiotherapy and Radioresistance of GB

### Radioresistance of GB

GB tumors have been identified as therapy resistant due to multiple molecular mechanisms including inadequate drug blood-brain barrier (BBB) passage, intra- and intertumoral heterogeneity, redundant signaling pathways resulting in rescue mechanisms, adaptive radioresistance and an immunosuppressive tumor micro-environment (TME) promoted by a chronic state of hypoxia (15, 42–44). Hypoxic niches limiting the formation of reactive oxygen species (ROS) and a hyperactivation of the DNA damage response machinery induced by glioma stem cells (GSC) contribute to glioma radioresistance (44, 45). In addition, a cross-talk between TME populations *via* shared pathways, such as STAT3, Wnt and Notch play a role (15, 46).

### New Developments in GB Radiation Therapy

Alternative RT technologies to improve therapy effectiveness in GB, including dose escalation, a stereotactic radiosurgery boost, brachytherapy and boron neutron capture therapy, have failed to become incorporated in the routine management of newly diagnosed malignant glioma (47, 48). However, several technological advances can contribute to a reduction of RT induced acute and late normal tissue toxicity. Three major examples are intensity-modulated radiotherapy (IMRT), proton therapy (PT) and ultra-high dose rate (FLASH) RT, which are promising to reduce cognitive impairments that could negatively impact the quality of life of GB survivors (49, 50). Compared to photon-based therapies, dosimetric PT studies in gliomas have shown a dose reduction to nearby organs at risk (OARs) and a lower risk of developing RT-induced tumors, which could even further improve with advanced intensity modulated proton therapy (IMPT) (14, 51–53). However, this is of less importance in GB compared to low-grade gliomas, due to the low median survival of GB patients. A phase II trial which compares PT with IMRT in their ability to preserve brain function in patients with IDH mutant grade II/III glioma is currently running (NCT03180502) (54). In conjunction with that, the outcome of PT dose-escalation and randomized clinical trials of PT versus IMRT are also currently under investigation (NCT01854554, NCT04752280, NCT02179086, NCT03180502) (54, 55).

Compared to PT, the unique physical and biological properties of high linear energy transfer (LET) radiation, such as carbon ion radiotherapy (CIRT), are expected to overcome microenvironmental limitations present in GB, such as hypoxia, and confer an improved glioma and GSC killing ability (56–58). In GSC models, CIRT showed to overcome glioma radioresistance by eradicating stem cells, inducing anti-angiogenic effects and influencing the immune system (42, 59, 60). For the treatment of brain tumors, multiple clinical studies have suggested that CIRT is effective with a favorable toxicity profile, mainly through the delivery of a carbon ion boost following conventional RT or PT (61, 62). This led to the prospective CLEOPATRA Trial at Heidelberg Ion Therapy Center (HIT) and a Phase I/III clinical by the Shanghai Proton and Heavy Ion Center (NCT04536649) (42, 48, 61, 63). First results applying particle RT at a dose ≥60 gray-equivalents showed to be safe and potentially effective with an 18-month overall survival (OS) rate of 72.8% and progression free survival (PFS) rate of 59.8% (48). CIRT is also being investigated in recurrent GB, with results of the randomized phase I/II CINDERELLA trial pending (64). For recurrent high-grade glioma, the recent study of Eberle et al. deemed carbon ion reirradiation as safe and feasible (65).

In FLASH RT, the dose is delivered at ≥ 40 Gy/sec compared to dose rates of approximately 1-4 Gy/min in conventional EBRT (66). This technique provided encouraging results in an *in vivo* study using a murine GB model, but is currently still limited to superficial tumors using electron beams (67). New developments in FLASH proton and heavy ion beam therapy look promising and could pave the way to treat deeper seated tumors in a clinical context, such as GB (68, 69). The combination of FLASH with mini-beams, could even further increase the protection of healthy tissue and preserve anti-tumoral immunological reactions (70).

## Role of the MDM2X-p53 Pathway in Cancer and GB

### The MDM2/X-p53 Pathway

**Figure 1:**
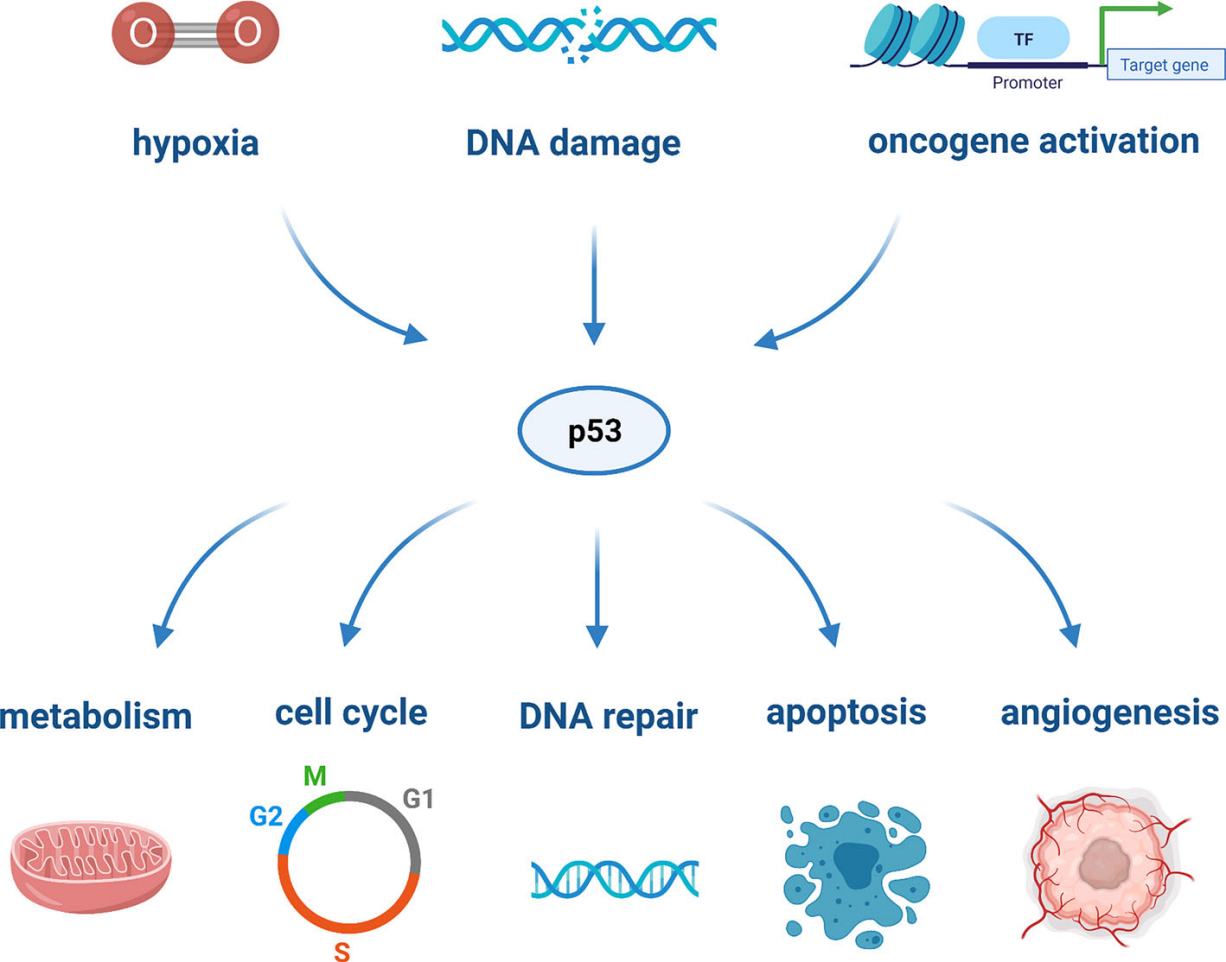
The various cellular processes regulated by p53 in response to cellular stressors.

TP53 is markedly the most studied tumor-suppressor gene. It encodes the tumor suppressor protein p53 which, in light of its nature and action, has been defined as the “guardian of the genome”. It is a multifunctional transcription factor that can be activated through cellular stresses, such as hypoxia, DNA damage, or oncogene activation. Upon activation, p53 acts as a tumor suppressor and responds to cellular damage by mediating cell proliferation, arrest, DNA repair, metabolism, angiogenesis, senescence and apoptosis, as depicted in **Figure 1** (20). The most critical downstream targets of p53 are the apoptotic proteins, as they are responsible for the activation of various cell death pathways (35). The activation of the latter plays a role in prohibiting the replication of damage-causing genetic lesions, as these could result in unconstrained cell growth and oncogenesis (71).

**Figure 2:**
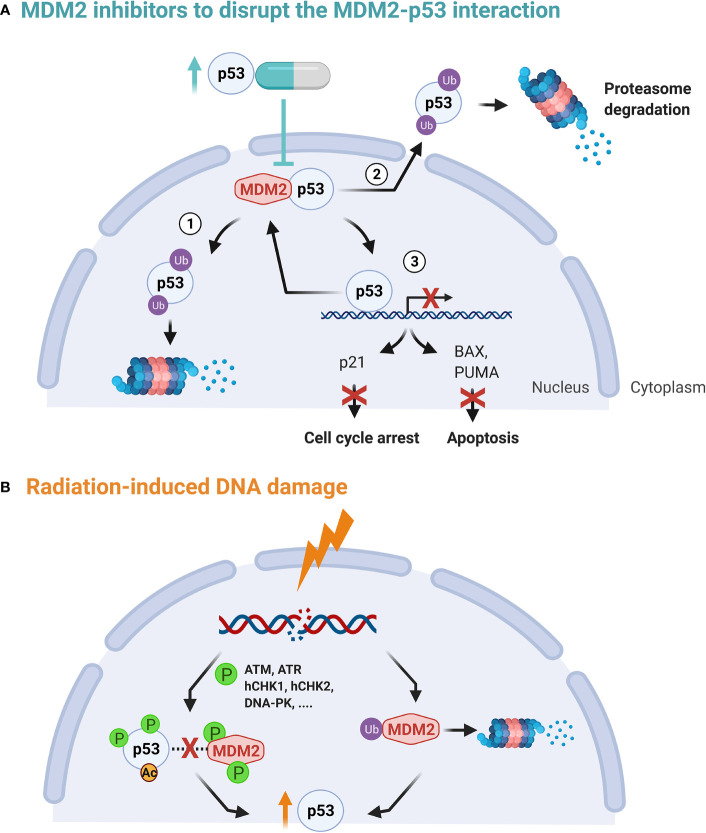
**(A)** The p53-MDM2 autoregulatory feedback loop. p53 stimulates MDM2 expression while MDM2, in turn, inhibits p53 activity by stimulating its degradation in the nucleus and the cytoplasm (1, 2), promoting its nuclear export (2) and blocking its transcriptional activity (3) (72, 73). **(B)** Upon DNA damage, both MDM2 auto-degradation and phosphorylation of p53 is activated. This in turn disrupts the MDM2 binding, increasing transcription activation and stability of the p53 protein. In addition, ATM phosphorylation of MDM2 is critical for MDM2 destabilization, leading to less p53 ubiquitination (74, 75).

In normal conditions and in the absence of cellular stress, cellular homeostasis is set to preserve low p53 levels. This level is regulated by MDM2, a E3 protein ligase which is responsible for p53 degradation through a ubiquitin-dependent pathway. When the amino-terminal domain of MDM2 binds to p53, the transcriptional activity of p53 is inhibited and the p53 protein complex is exported from the nucleus to the cytoplasm for degradation by cytoplasmic proteasomes. In this way, both the p53-mediated cell cycle arrest and the apoptosis functions of p53 are affected (**Figure 2A**) (72, 73). Hence, targeting the interaction between p53 and the E3 ligase MDM2 represents an attractive anti-cancer approach with the condition that the tumor is wild-type (*wt*) TP53 or functional TP53 is present (40). The p53-MDM2 pathway is also referred to as the p53-ARF-MDM2 pathway, since ARF (alternative reading frame), is a tumor suppressor that interacts with MDM2. This interaction prevents MDM2 shuttling between the nucleus and cytoplasm and thereby circumvents p53 degradation (76).

Secondly, upon sensing DNA damage, ataxia telangiectasia mutated (ATM) becomes activated and induces phosphorylation of p53 and MDM2 directly or indirectly *via* checkpoint kinases, such as hCHK1 and hCHK2. The latter prevents their interaction and guarantees the stabilization of p53, see **Figure 2B** (72, 77). DNA damage has also shown to induce MDM2 auto-degradation (78). However, high levels of p53 in their turn activate transcription of downstream targets, including MDM2. Hence, the above mechanisms form a autoregulatory loop to control the amount of p53 and MDM2 proteins (74, 79, 80).

The MDM2 homologue protein MDMX (also known as MDM4) shares some similarity with MDM2 in the p53 binding domains, but they are not identical and MDMX exhibits no E3 ligase activity. MDMX is able to inactivate p53 in two ways: by binding to the N-terminus of p53 directly or by heterodimerization with MDM2 stimulating its ubiquitination function. This is called the p53-MDM2/MDMX loop, in which both MDM2 and MDMX act as inhibitors of p53’s tumor suppressor function (81, 82). At variance to MDM2, MDMX appears not to be transcriptionally regulated by p53, as explained by Marine et al. (83).

### The Role of the MDM2/X-p53 Pathway in GB

Alterations of the p53 pathway are common in multiple cancer types, including GB. It is clear that the most common cause for TP53 deregulation is due to MDM2 and MDMX amplification as well as missense mutations in the TP53 gene, which results in the demise of its role as a tumor suppressor. This area has been extensively reviewed by Zhang et al. (20). The complicated genetic profile of GB was confirmed by genomic profiling and the Cancer Genome Atlas project, which revealed a set of three core signaling pathways that are commonly altered in GB: the p53 pathway, the receptor tyrosine kinase/Ras/phosphoinositide 3-kinase (PI3K) signaling pathway, and the retinoblastoma (Rb) pathway (74, 77, 84, 85). Alterations of the p53 pathway play a key role in GB development, cell invasion, migration, proliferation, apoptosis, cancer cell stemness and resistance to TMZ treatment (86–88). Interestingly, genomic characterization of human GB genes and its core pathways showed that p53 signaling was altered in 87% of GB cases (84). More specifically, 84% of GB patients and 94% of GB cell lines showed a deregulation of the p53-ARF-MDM2 pathway (4, 20). In primary GBs, TP53 is relatively infrequently mutated (25-30%), while in secondary GB, alterations of p53 are observed in 60-70% of cases (89). The prevalence of TP53 mutations also depends on the GB molecular subtype: proneural (54%), mesenchymal (32%), neural (21%) and classical (0%) respectively  (20, 90). However, even in p53 *wt* GB, p53 availability is frequently reduced because of interactions with overexpressed MDM2 proteins (86–88). An amplification and overexpression of MDM2 gene is observed in 14% of GB cases (84). Concerning MDMX, a 5- to 25-fold amplification in 2.4% of 208 glioma cases was assessed by Riemenschneider et al. and interestingly, all had a retained p53 *wt* status. Of these, none showed MDM2 amplification (91). Another study performed qPCR on 86 GB samples and found an amplification of the MDMX gene in 27% of these samples. They also observed a 28.6% MDMX amplification of low-grade astrocytic tumors and deduced that this could signify an early event in carcinogenesis (92, 93). Hence, reactivating p53 activity through inhibition of MDM2/X offers a tenable opportunity for therapeutic intervention in GB.

## MDM2 Inhibitors as an Anti-Cancer Strategy

As previously mentioned, TP53 function can also be suppressed in p53 *wt* tumors *via* MDM2 overexpression, limiting the p53 protein to perform its tumor suppressor role and thereby promoting cancer progression (94, 95). As such, the re-activation of the p53 pathway is regarded as a plausible anti-cancer strategy and has the potential to increase the radiosensitivity of cancer cells. The main p53-based targeted therapies involve either the use of targeted molecules to convert the mutant (*mut*) p53 back to its *wt* form or MDM2 inhibitors which allow tumors with a p53 *wt* form but with MDM2 amplification to consequently restore p53 functioning (35, 71, 96).

One of the first attempts at understanding the mechanisms behind p53 reactivation entailed the phosphorylation and acetylation of its complex. Studies revealed that although the latter plays a role in weakening the p53-MDM2 interaction, it is not critical for p53 stabilization upon DNA damage (72). Consequently, the MDM2 protein itself became the principal target. Since the structure of the MDM2-p53 interaction has been revealed, multiple SM MDM2 inhibitors have been developed against the p53-binding pockets of MDM2 (95). These include nutlins, spiro-oxindole derivatives and piperidinone-containing compounds, such as MI-77301/SAR405838, APG-115, MK-8242, RG7112, RG7388, DS-3032b, and AMG232. An overview of different categories of MDM2 inhibitors, their design and the current status in the clinic has been reviewed elsewhere (31, 32, 34, 41, 74). Peptides have also been studied as potent inhibitors of the p53-MDM2 interaction and a number of these induced p53 mediated cell cycle arrest and apoptosis in solid cancers and hematological malignancies (88, 97–100). However, it is important to note that tumors harboring p53 mutations are not responsive in contrast to p53 *wt* tumors. Furthermore, sensitivity to MDM2 targeted therapy increases when p53 *wt* tumors also show MDM2 amplification (29, 98, 99). Clinical trials on MDM2 inhibitors are ongoing in acute myeloid leukemia (AML) (NCT02319369, NCT03634228), soft tissue sarcoma (NCT03217266), malignant salivary gland carcinoma (NCT03781986), pediatric cancers (NCT03654716) and small cell lung cancer (NCT04022876) (51, 52).

Additionally, MDMX antagonists have shown to inhibit the MDMX-p53 interaction. As an example, Pellegrino et al. identified a peptide that mimics the MDMX C-terminus, and binds MDM2, thereby blocking the MDMX/MDM2 complex (101). Importantly, the amount of MDMX influences the sensitivity to MDM2 inhibitors and the susceptibility to MDMX targeting appears to be dependent on the levels of p53 and especially of MDM2 (102, 103). Hence, studies have shown that combination therapy using MDM2/MDMX inhibitors result in a more effective anti-tumor reaction by more actively inducing apoptosis and cancer cell cycle arrest (81, 101, 104). For more extensive reviews on targeting MDM2 and MDMX in cancer therapy, see (30, 37, 74, 102, 105–107).

Two tumor characteristics enable a selection of patients who could benefit from MDM2- and MDMX-based therapies aimed at reactivating p53 function: a p53 *wt* status and overexpression of MDM2, MDMX or both. In addition, through the understanding of the dysregulation and functioning of MDM2 and MDMX in GB cancers, diagnostic and prognostic methods could be improved for a more personalized approach (29).

## MDM2 Inhibitors as Radiosensitizers

### Rationale for the Radiosensitizing Effect of MDM2 Inhibition

The concept behind radiosensitizers is based on their ability to enhance the radiosensitivity of cancer cells, resulting in increased radiation-induced cell killing. This can be achieved by targeting specific radiation response mechanisms, such as DNA repair mechanisms, and in the case of MDM2/X inhibitors, the p53 transcription factor pathway (18, 82). The actions of p53 are critical in determining the effectiveness of IR and/or chemotherapeutic agents (79). The cellular effects induced by IR are mediated by the DNA damage response (DDR) pathway, which facilitates MDM2-p53 signaling *via* activated kinases, such as ATM (see **Figure 2**). In cancer cells with p53 *wt* genes, the level of both MDM2 and p53 expression is directly correlated to the amount of IR induced DNA damage. Radiosensitive tissues have shown prolonged p53 signaling after IR, while more resistant tissues show transient p53 activation (108). Within the two major pathways in DNA double-strand break (DSB) repair, p53 interacts with both non-homologous end-joining (NHEJ) proteins as well as with protein RAD51 which plays a major role in homologous recombination (HR), influencing their expression (82, 109).

The effectiveness of IR to treat cancer is hampered by MDM2 mediated p53 inhibition, causing a decrease in DNA damage cell cycle arrest and apoptosis (110). As a result, MDM2 overexpression has been correlated with a decreased therapeutic response and failure of p53 to induce p21^BAX^ expression has been linked to radioresistance in GB cells (79, 111). Blocking of the negative regulators MDM2 and MDMX could be a promising strategy to improve RT outcomes of *wt* TP53 GB - see **Figures 2A, B**. Sustaining p53 using MDM2/X inhibitors has shown radiosensitizing effects pre-clinically in lung cancer, prostate cancer, adenocarcinoma and colon cancer (21, 82, 108, 110, 112, 113). Remarkably, glioma cells lacking p53 *wt* function seem to be susceptible to IR-induced apoptosis due to an increased caspase-8 activity, which may be triggered by ceramide (114, 115).

Some critical factors will have to be considered when MDM2/X inhibition is combined with IR. Firstly, the effects on non-cancerous (brain) tissue have been poorly researched. Different cells/tissues can show different levels of apoptotic response to IR and the restoration of p53 in non-cancerous tissues levels after non-lethal DNA damage should take place rapidly to avoid unnecessary cell death. MDM2 inhibitors could however promote cell cycle arrest in non-cancerous cells and tissues that surround the tumor, without affecting tumor cells in case the tumor is p53 *mut*. However, the toxicity to healthy tissues might be limited since MDM2 inhibitors, such as nutlins (MI-219), have shown to activate p53 in normal tissues with limited p53 accumulation in contrast to a robust accumulation of p53 in normal tissues induced by chemo/radiotherapy (94, 116). An optimal approach would be to influence the dynamics of p53 differently between tumor and normal tissues following genotoxic therapies (108). Secondly, MDM2 has been reported to have p53-independent functions, also influencing the cell cycle, and DNA repair, amongst others (117). Particularly the interaction between MDM2 and the DNA repair complex (Mre11/Rad50/NBS1 or MRN) at DNA damaged sites is important concerning the response to IR. Nbs1 has been identified as a p53-independent MDM2 binding protein. This interaction in turn reduces DNA damage signaling levels and causes significant delays in DNA break repair, which might be an important side effect to take into consideration in the normal tissue response (118, 119).

For the aforementioned reasons, the synergy between MDM2 inhibitors combined with IR exposure may offer a more effective cancer treatment strategy, but more research is needed to reveal the exact mechanism of action and possible normal tissue toxicities (72). Two aspects must be considered: 1) MDM2 inhibitors may not be effective in GB tumors with inactivation of p53, 2) MDM2 inhibition combined with IR may lead to the radiosensitization of normal tissues (74, 116). Therefore, the targeted delivery of MDM2 inhibitors is crucial to induce targeted apoptosis of cancer cells and limit toxicity in normal tissues.

### Activating the p53 Pathway in Combination With Different Radiation Qualities

Different apoptotic signaling mechanisms and p53 dependency have been suggested between different radiation qualities (120–124). For increasing LET a tendency towards an increased apoptotic response has been observed (121, 125, 126). In normal human fibroblasts, the induction of TP53 and CDKN1A was dependent on the dose and LET (123). Also, p53 was slightly induced by both proton and X-ray irradiation, while a significant increase in protein expression of a downstream regulator of p53, CDKN1A, was seen after low-energy proton irradiation (127). A greater TP53 protein accumulation was observed after carbon ion exposure, compared to that of iso-doses of X-rays (123). In GB cell lines, X-rays, CIRT or alpha-particle IR all induced p53-dependent p21 accumulation (128).

Compared with photon radiation, PT has shown to induce more robust DNA damage and reduced cell cycle recovery from G2 arrest, leading to apoptosis and cytotoxicity (127, 129, 130). In addition, the mechanism of cell death induced by high LET CIRT is significantly different when compared to low LET radiation. This includes a greater ability of inducing the ceramide pathway and more complex DNA DSB damage resulting in increased levels of autophagy and apoptosis (131–133). High LET radiation phosphorylated p53 at serine 37, which is involved in cell death, more extensively compared to low LET irradiation (134). Different amounts of ROS induced by different radiation qualities will also impact the activation of p53, which can in turn activate cell survival and/or cell death processes (135).

Importantly, the presence of p53 seems to be crucial for the induction of apoptosis by PT, while the induction of apoptosis by high LET (in the order of 70 keV/µm) radiation, such as iron ions, was seen regardless of TP53 gene status in cancer cells (122, 124). Instead, in case of high LET radiation, caspase-9 activation plays a role in apoptosis enhancement in mutated p53 cancer cells and suppression of AKT (serine/threonine protein kinase B)-related signaling inhibits cell growth (122, 136). The response of GB cells to photon and CIRT irradiation also included an p53 independent G2/M phase arrest and subsequent appearance of mitotic catastrophe, while a ceramide-dependent-apoptotic cell death was observed (131). However, studies on p53 targeted drugs, such as MDM2 inhibitors, and the potential differences in radiosensitizing effects for different radiation qualities remain limited.

### MDM2/X Inhibition Combined With Irradiation for GB Therapy

In non-GB cancer types, preclinical evidence has been provided of a RT sensitization effect induced by MDM2 inhibitors, including nutlins, serdemetan/JNJ-26854165, APG-115, PM2 and MI-219 (21, 112, 113, 116, 137–141). Interestingly, data showed that Nutlin-3 acted as a radiosensitizer under hypoxic conditions and as a radiosensitizer of tumor vasculature (140, 141). One of the main conclusions of this literature review is the fact that the combined strategy of MDM2/X inhibitors with RT is underexplored for GB. In p53 *wt* glioma cell lines, an enhanced radiosensitivity was observed when Nutlin-3 was combined with X-rays (142). RG7388 and RT also showed synergism, however, long-term treatment induces resistance (29). The RG7388/RT combination is also included in a phase I/IIa trial in patients with newly diagnosed GB without O(6)-methylguanine-DNA methyltransferase (MGMT) promotor methylation (N²M² (NOA-20), NCT03158389). Nutraceutical resveratrol, which has been reported to induce p53 and its downstream targets, acted as a radiosensitizing anticancer agent for highly radioresistant human SU-2 GSC both *in vitro* and *in vivo* (143, 144).

## Current Status of MDM2/X Inhibition and p53 Activation for the Treatment of GB

Despite the limited studies performed on the combination of IR and MDM2/X inhibitors for the treatment of GB, this section will give an extensive overview of all GB studies investigating MDM2/X inhibitors and other approaches to activate p53 (**Table 1**).

**Table 1:** Overview of single or combined GB treatment strategies with MDM2/X inhibitors.

	Treatment	Type	C/PC	GB *in vitro* / *in vivo* model	Results**	Reference
**MDM2/X inhibition combined with irradiation**						
*MDM2 - RT*	Nutlin-3 + X-rays (0, 2, 4, 6, 8 Gy)	SM	PC	U87MG *wt*, T98G *mut*	Varying levels of apoptosis and senescence and an enhanced radiosensitivity among the different p53 *wt* GB cell lines. GB cell lines with mutated or knockdown p53 were completely unresponsive to the drug	(142)
	Resveratrol + X-rays (2, 4, 6 Gy)	Na	PC	SU-2 GSCs	Radiosentizing effect on GSCs. The combination has synergistic antitumor properties like blockade of proliferation, triggering of autophagy, facilitation of apoptosis as well as preclusion of DNA repair	(145)
	RG7388**^£^**	SM	C	GB patients with an unmethylated MGMT promoter	Included in active N²M² (NOA-20) trial in in conjunction with RT	NCT03158389 (54)
			PC			
				U87MG *wt*	Combination with RT showed inhibited clonogenicity. Induced cell cycle arrest and apoptosis. However, long-term treatment induces resistance to treatment (2Gy and 4Gy)	(29)
**Targeting the MDM2-p53 interaction**						
*Nutlins*	Nutlin-3a	SM	PC	NOD.Cg-Prkdc^scid^IL2rg^tm1Wjl/Sz^ (NSG) mice	Three cycles of TMZ/nutlin3a**** resulted in a significant survival increase of the GB10 intracranial *in vivo* model compared with single therapy	(146)
	RG7112	SM	PC	SJ-GBM2, GBM2, BT-39, D645, D456, CB17SC scid -/- female mice	Reduced tumor growth in GB PPTP^&^ models *in vitro* and *in vivo*	(23)
			PC	U373MG *mut*, LN18 *mut*, U251MG *mut*, A120*wt* ^T^, DBTRG-05MG*wt*, U87MG *wt*.	A greater sensitivity of *wt* cell lines were observed, while the mutant p53 cell lines showed resistance	(26)
			PC	U251MG *mut*, U87MG *wt* LN229 *mut*	Restored p53 activity inducing strong p21 expression and apoptosis. PK profiling demonstrated crossing of the BBB. Cytotoxicity was observed, but treatment reduced tumor growth and increased survival.	(147)
	RG7388	SM	C, PC		See**^£^**	NCT03158389 (29)
*Piperidinones*	AMG232 (KRT-232)	SM	C	Recurrent or newly diagnosed GB	Included in active N²M² (NOA-20) trial in conjunction with RT and a phase I trial	NCT03158389 (54)
			PC	U373 *mut*, LN18 *mut*, U251 *mut*, A1207*wt*, DBTRG-05MG*wt*, U87MG *wt*	9.5-fold more effective than RG7112 in p53 *wt* GB cells	NCT03107780 (54)
				10 patient-derived GSCs	MDM2-amplified stem cells (464T) were 35-fold more sensitive to AMG232	(148)
				100 patient derived GB cell cultures, with computational modelling	Potentiated the effect of bortezomib in multiple GB cell lines by increasing apoptotic effects	(26)
*Spirooxindole Derivatives*	ISA27	SM	PC	U87MG *wt*	Synergy with TMZ: effective in inhibiting cell growth, to such an extent to possibly lower the dose of TMZ	(88)
	Spiropyrazoline oxindole 1a	SM	PC	GL-261	Treatment showed a decrease in SOX2 protein levels, thereby reducing stemness. In addition, chemotherapy sensitization in combination with TMZ was observed	(149)
	MI77301 (SAR405838)	SM	PC	PDX models of GB	A sensitivity was observed in MDM2-amplified PDX lines with high MDM2 expression in comparison to MDM2 control lines in both *in vitro* and heterotopic models. Contradictory results for orthotopic tumors: inefficiency	(28)
*Other*	MK-8242 (formerly SCH 900242)	SM	PC	PPTP^&^ cell line panel including GB cell lines SJ-GBM2, GBM2, BT-39, D645, D456	Cell lines with *wt* TP53 showed a sensitivity, while a resistance for cell lines with *mut* TP53 was observed. Results showed a reduction in tumor growth for most of the PPTP^&^ panel as well as the xenograft models	(25)
**Other approaches to enhance p53 activity in GB**						
*Blocking MDM2 expression*	SP-141	SM	PC	U87MG, SNB19, U251, LN229, T98G, GBM10, SF188, UW18 and UW28 cell lines	Effectively induced cell cycle arrest and apoptosis. Effective antitumor activity against U87MG intracranial xenografts and combination treatment with TMZ resulted in more effective cell killing and suggested to aid in TMZ resistance	(146)
	miR-129	miRNA	PC	U251 *mut* and U87MG *wt*	rtPCR done on cell lines significantly reduced the expression of MDM2, resulting in cell cycle arrest	(150)
	miR-17	miRNA	PC	U87MG *wt*	Repressed MDM2, resulting in decreased cell proliferation and drug resistance	(151)
	miR-4486	miRNA	PC	Glioma cells - U87MG, U251, SHG-44, SW-38	Down-regulation of MDM2 by miR-4486 increased the abundance of p53 in glioma cells	(152)
*Restoration p53 expression or active conformation*	CP-31398	SM	PC	LN-18, U138MG, U87MG, LN-428, D247MG, T98G, LN-319, LN-229, A172, U251MG, U373MG, LN-308	p53 reporter gene activity in all of tested glioma cell lines harboring either *wt* or *mut* p53 was induced. All cell lines underwent a caspase-independent and bcl-xL-insensitive cell death after prolonged incubation	(153)
	PRIMA-1	SM	PC	Multiple p53 *mut* GB cell lines	Despite showing selective single agent activity in p53 *mut* cells, it did not increase bortezomib activity	(154)
				GB mouse models	Restores p53 *wt* conformation by altering p53 *mut* protein folding - inhibition of cell growth and stemness as well as apoptosis induction	(19)
	NSC319726	SM	PC	GB patient derived cells	Induces copper-dependent cell cycle arrest at picomolar concentrations	(155)
	RITA	SM	PC	U251 *mut* and U87MG *wt*	Inhibited proliferation of p53 *mut* U251 more effectively than p53 *wt* U87MG GB cell lines	(156)
	P53R3	SM	PC	T98G, U251, U373MG, U138MG, LNT-229	Restored p53 expression and induced antiproliferative effects, resulting in a higher apoptotic induction rate	(157)
	p53p-Ant	P	PC	Human: U138, U87MG, Rat: 9L, D74, F98, NL	A 3-fold increase in extracellular membrane Fas expression, resulting in activation of p53 function and consequently induction of apoptosis in both p53 *mut* and *wt* cell lines	(158)
	SGT-53 gene therapy	Nanocomplex that delivers p53 *wt*	PC	GL261	Enhanced anti-tumor effects and reduced tumor cell proliferation	(159)
	Retroviral-mediated gene transfer	GT	PC	U87MG *wt*	Retroviral-mediated gene transfer of the p53 (175H) *mut* promotes apoptosis in association with adenoviral-mediated p53 *wt* gene transfer	(160)
	CRAd**^#^**AdDelta24-p53 + RT	GT (adenovirus)	PC	glioma cells *in vitro* and *in vivo*	Combination of RT and AdDelta24-p53 caused an increase in apoptosis. *In vivo*, combination therapy increased tumor regression and long-term survival	(161)
	p53-NLS-Ln-11R**^%^**	P	PC	glioma cells – YKG1 *mut*, T98G *mut*, U87MG *wt*	This protein-transduction method inhibited the proliferation of human glioma cells, whether the p53 gene had mutated or not	(162)
*Influencing MDM2-proteasome interaction*	JNJ-26854165 (Serdematan)	SM	PC	SJ-GBM2	Shows activity against both p53 *wt* and p53 *mut* cell lines and xenografts, including GB	(163)
*Inhibition of the E3 ubiquitin ligase activity of MDM2*	USP2a	Ubiquitin-specific protease 2a	PC	U87MG *wt*	Results suggest that USP2a binds to and stabilizes MDMX, with subsequent higher mitochondrial localization of p53 and apoptosis	(164)
*Natural compounds*	Curcumin	Na	PC	SH-SY5Y neuroblastoma	Inhibits cell growth, arrests cells at S phase and induces apoptosis by decreasing the MDM2 protein level	(165)
				U87MG *wt* xenograft	Increased cell death, reduced cell growth and inhibited migration and invasiveness	(166)
				U251	Inhibited cell growth and induced G2/M and S-phase arrest in a dose dependent manner	(167)
	Flavopiridol	Na	PC	A172, CCF-STTG1, T98G, U87MG, U118MG, U251MG, and U373MG	Inhibited cell growth, arrested cells at G2/M phase and induced apoptosis by decreasing the MDM2 expression at mRNA level	(168)
	Chalcone	Na	PC	U87MG *wt* cells and xenograft	Inhibits cell growth, arrested cells at G1 phase and induces apoptosis by decreasing the MDM2 protein level. Inhibited tumor growth in U87MG xenograft mouse model	(165)
	Resveratrol	Na	PC	U87MG *wt* cells	Activates transcription of downstream p53 targeted genes, which leads to a decreased affinity for MDM2, causing an increase in p53 stability and thereby cell cycle arrest and apoptosis	(144)
**Dual MDM2/MDMX inhibitors**						
*Peptide based MDM2/MDMX inhibitors*	D-PMI-beta	P	PC	U251 *mut* and U87MG *wt*	Works in a p53-dependent manner as U251 mutated cells were resistant to treatment and successful growth inhibition was observed in U87MG *wt* cell lines	(97)
	liposome-PMI1-4	P	PC	U87MG *wt* and U251	PMI failed to inhibit cell growth through MDM2/MDMX targeting. Peptide-loaded liposomes were designed to improve cellular uptake of the drug. Liposome-PMI-1 was the most effective in inducing apoptosis of U87MG cells, but not U251, indicating a p53 dependent interaction	(169)
	PM2	P	PC	4-10 GB cell lines	Potentiated the effect of the protease inhibitor bortezomib in multiple GB cell lines by effectively inducing cell death after treatment	(154)
	RGD-M/sPM**^€^**	RGD-peptide micelle	PC	U251 *mut* and U87MG *wt*	RGD-liposomal pDP treatment increased the median survival time of intracranial U87MG GB nude mice. Western blot assay validated the reactivation of p53 through MDM2 inhibition in both cell lines	(170)
*Other*	NSC623731	SM	PC	U87MG *wt*	Demonstrated to possess anti-proliferative activity	(171)
**MDM2/X inhibition and other combined treatment strategies**						
*MDM2-chemotherapy*	Nutlin-3a + Doxorubicin	SM	PC	U87MG *wt*	Treatment resulted in the reactivation of the p53 pathway, leading to an increase in p53 activity and consequently sensitization of the GB cells	(172)
	Spiropyrazoline oxindole 1a + TMZ	SM	PC	GL-261	Chemotherapy sensitization in combination with TMZ	(149)
	RITA + TMZ	SM	PC	U251 *mut* and U87MG *wt*	Inhibited proliferation of p53 *mut* U251 more effectively than p53 *wt* U87MG GB cell lines. In both instances, apoptosis was induced more effectively in combination with TMZ	(156)
	RGD-M/sPM**^€^** + TMZ	RGD-peptide micelle	PC	U87MG *wt*	Anti-glioma effect through activation of the p53 pathway *in vitro* and i*n vivo*. Synergistic with TMZ	(170)
	Resveratrol + TMZ	Na	PC	Human GB-initiating cells	Enhanced the sensitivity to TMZ via activation of the DSB/ATM/ATR/p53 pathway, leading to the activation of apoptosis	(173)
	RG7112 siRNA	SM siRNA	PC	U87MG *wt* cells and *in vivo DK-MG (p53 wt), LN308 (p53 null), and U251 (p53 mut)*	Enhanced the sensitivity to TMZ, reversing the YB-1 protein mediated TMZ drug resistance	(174)
*MDM2-integrins*	Compound 9	Pe	PC	U87MG *wt cells*	Effective in inducing long term cell cycle and proliferation arrest of GB cells by targeting MDM2/X as well as α5β1/αvβ3 integrins	(175)
*MDM2-Akt/mTOR*	FC85 +ISA27	SM	PC	U87MG *wt* cells	Synergic effect on the inhibition of cell viability and on the reactivation of p53 pathway. Also blocked proliferation and promoted the differentiation of GSCs	(176)
*MDM2-CDK4*	Ent-4g*	S	PC	T98G *mut*, U251 *mut*, U87MG *wt*	Induced apoptosis and cell cycle arrest. Cells treated showed up-regulation of proteins involved in P53 and cell cycle pathways. Anti-tumor efficacy against GB xenografts in mice	(177)
*MDM2-MEK*	RG7388 + Trametinib	SM	PC	U87MG, A172, T98G, LN428, LN308 and LN229; Xenograft mouse model	Clonogenicity synergistically inhibited through the combination, resulting in a restored sensitivity towards RG7388 in U87MG and A172 cell lines. *In vivo*, results demonstrated a reduction of tumor growth	(29)
*MDM2/X-CXCRX*	RS3594 + AMD3100	SM	PC	Human GB cells and GB stem-like cells (neurospheres) U87MG, T98G, U343MG	Reduced GB cell invasiveness and migration in single agent treatment but this increased in the combined treatment regimen with synergic effects on cancer stem components.	(178)
*MDM2/V-ATPase*	Nutlin-3a + V-ATPase inhibitor (archazolid)	SM	PC	U87MG *wt*	Synergistic for inducing cell death in different p53 *wt* tumor cell lines and highly activated pro‐apoptotic pathways. Combination is more efficient in reducing tumor growth compared to single treatment *in vivo*	(179)
*Other*	p19Arf gene transfer and nutlin-3	SM	PC	C6 *wt* GB cell line	C6 cells were quite susceptible to both, yet p53 was further activated by the combination. Results showed a marked increase in cell cycle alterations and an increase in p53 activity, thereby resulting in cell death	(180)

ataxia telangiectasia mutated (ATM), ataxia telangiectasia and Rad-3 related (ATR), BBB (blood brain barrier), BTSC (patient-derived brain tumor stem cell), C (clinical study), DSB (DNA double strand break), GSC (glioma stem cells), GT (gene transfer), MGMT [O(6)-methylguanine-DNA methyltransferase], P (Peptides), PC (pre-clinical study), PDX (patient-derived xenograft), Pe (Peptidomimetics), S (spirooxindoles), (Pi) Piperidones, miR (microRNA), Na (Natural compounds), NSG (NOD scid gamma mouse), SM (small molecule), RT (radiation therapy), TMZ (temozolomide), ^€^RGD-M/sPMI [cyclic RGD peptide-conjugated poly (ethylene glycol)-co-poly (lactic acid) polymeric micelle (RGD-M) that carried a stapled peptide antagonist of both MDM2 and MDMX (sPMI)], ^&^Pediatric preclinical testing program (PPTP), ^%^p53-NLS-Ln-11R (polyarginine11R as a PTD, nuclear localization sequence (NLS), and laminin (Ln) fused to the p53 peptide corresponding to the MDM2 binding site), **^#^**conditionally replicating adenovirus (CRAd), *tetrahydronaphthalene fused spirooxindol.

### Targeting the MDM2-p53 Interaction

#### Nutlins

Nutlin-3**** is the first potent MDM2 SM inhibitor that was identified (181). Its analogue Nutlin-3a was effective at inhibiting GB cell growth, inducing varying levels of apoptosis and senescence, decreasing TMZ resistance and acting as a radiosensitizer (88, 142). The first modified MDM2 inhibitor that reached clinical trials was a more potent Nutlin analogue ****RG7112 (182). RG7112 showed a potential cell killing effect in GB both *in vitro* and *in vivo*, with up to a 44 times higher efficacy in MDM2-amplified and p53 *wt* GB cell lines (147, 183). In several Phase I trials in solid and hematological malignancies, RG7112 was successful in activating p53 and subsequently increasing the expression of downstream pro-apoptotic proteins. However, the higher dose that was required to attain satisfactory p53 activation caused significant toxicities (184–187). A second-generation nutlin analogue, ****RG7388 (idasanutlin), showed an increased potency, selectivity, and had a better pharmacokinetic profile. This SM inhibitor has been studied in both solid and hematological malignancies (188–190). RG7388 is included in the N²M² (NOA-20) trial in conjunction with RT with the aim to increase OS of patients with GB with an unmethylated MGMT promoter status (NCT03158389) (54).

#### Piperidinones

After nutlins, piperidinone-based compounds were identified as potent MDM2-p53 interaction inhibitors. Their discovery and development for targeted cancer therapy has been reviewed elsewhere (34). AMG232**** consists of a piperidinone scaffold which is similar to that of nutlins. AMG232, as a single therapy or in a combined treatment strategy, is under clinical evaluation for the treatment of advanced solid tumors, metastatic melanoma, multiple myeloma, soft tissue sarcoma and AML. At the moment, one clinical phase I trial is running in primary and recurrent GB (NCT03107780) (30, 54). In a phase I trial in p53 *wt* solid tumors which included GB, AML and multiple myeloma patients, AMG232 showed an acceptable patient tolerability and safety and favorable dose-proportional pharmacokinetics (191). AMG232 has also shown to increase the radiation response in several *in vitro* and *in vivo* experiments across a variety of p53 *wt* tumor types, but this was not studied in GB (21). However, it has been observed that AMG232 inhibition is more specific and highly regulated compared to RG7112 and its effect on GSCs was more potent (26, 148).

#### Spirooxindole Derivatives

ISA27**** has a spirooxoindolepyrrolidine core structure that has the ability to reactivate the antitumor capacities of p53 in GB cells by dissociating the MDM2-p53 complex. It has been shown to be non-toxic and it inhibited the growth of GB U87MG cells, with the implication that a lowering of the dose of TMZ as part of a combination therapy was suggested (88). The modified compound spiropyrazoline oxindole 1a**** was tested on the glioma cell line GL-261, alone and in combination with TMZ. These studies revealed an effective reduction in stemness through the reduction of the SOX2 protein levels, thereby promoting chemotherapy sensitization (149). Other spirooxindoles entered clinical trials and have been or are being studied in patients with advanced solid tumors and AML (****MI77301(SAR405838), DS-3032b/milademetan, APG-115) (27, 54). In patient-derived xenograft (PDX) models of GB, the effectiveness of MI77301(SAR405838) was dependent on MDM2 expression but limited by poor distribution across the BBB (28). In a phase I study in patients with advanced solid tumors, MI77301 had an acceptable safety profile but had limited single agent activity (54, 192). Pre-clinically, other spirooxindoles are currently being evaluated, such as MI-219, MI-63, MI-319, MI-43, MI-88, MI-137,**** but none of them include GB results (32).

#### Others

Novartis (Basel, Switzerland) designed a new category of MDM2 antagonists based on the dihydroisoquinolinone core which are being tested in clinical trials. These include CGM097**** and HDM-201**** (siremadlin) (38, 193, 194). A phase I study of CGM097 and HDM-201 in adult patients with selected advanced solid tumors was recently completed (NCT01760525, NCT02143635) (54, 195). Another SM inhibitor of the MDM2-p53 interaction, MK-8242**** (SCH-900242), has been investigated in a phase I trial in patients with advanced p53 *wt* solid tumors and AML. An acceptable safety and tolerability was shown after MK-8242 treatment, with a successful activation of the p53 pathway (196). In GB, data is limited, but the compound was included in the *in vitro* pediatric preclinical testing program (PPTP) that included GB and proved to be effective in reducing tumor growth by inhibiting MDM2 expression (25).

### Other Approaches to Enhance p53 Activity in GB

Next to blocking the interaction between MDM2 and p53, other strategies have been studied in GB to enhance p53 activity: blocking MDM2 expression, restoring p53 expression or its active conformation, influencing the MDM2-proteasome interaction and inhibiting MDM2 ubiquitin ligase activity (197).

#### Blocking MDM2 Expression

*In vitro* effects of the novel brain-penetrating SM MDM2 degrader SP-141**** was assessed on numerous GB cell lines. Binding of SP-141 to MDM2, induces MDM2 auto-ubiquitination and proteasomal degradation and inhibits its expression (146). Because SP-141 crosses the BBB adequately and due to its ability to eliminate MDM2 irrespective of the p53 gene status, this compound gained interest as a GB therapy agent (146, 198). Treatment *in vitro* resulted in a marked decrease of MDM2 and increase in p53 as well as G2/M cell cycle arrest and apoptosis. The inhibition of brain tumor growth by SP-141 therapy was confirmed *in vivo* and the combination with TMZ showed a synergistic cell death ratio (146).

Small interfering RNA (siRNA) and microRNA (miRNA) are other possibilities to influence MDM2 expression (150, 174). The miRNA precursor miR-129**** significantly reduced MDM2 expression in glioma cell lines, resulting in cell cycle arrest (150). miR-126**** expression is abnormally low in glioma cells and miR-126 inhibits the course of glioma through targeted regulation of phosphatase and tensin homolog (PTEN)/PI3K/AKT and MDM2-p53 pathways, which, therefore, can be used as a new potential biomarker (199). miR-4486**** has also shown to target MDM2 expression and increased the abundance of p53 in glioma cells (152). ****miR-17 transfected GB cells also showed a down-regulation of MDM2 expression, which resulted in an effective decrease in drug resistance and cell proliferation (151).

#### Restoration of p53 Expression or Active Conformation

The current approaches for (re)activating p53’s tumor suppressor role using SMs were recently reviewed by Silva et al. (200). In GB, stabilizing the active conformation of p53 by altering mutant p53 protein folding, has been explored with the SMs CP-31398, PRIMA-1, P53R3, NSC319726**** and RITA**** (Reactivation of p53 and Induction of Tumor cell Apoptosis). CP-31398 induced p53 reporter gene activity in all of the tested p53 *wt* and mutated glioma cell lines. High concentrations of CP-31398 resulted in the reduction of MDM2 mRNA expression (153). In GB cells, PRIMA-1 induces an inhibition of cell growth and stemness as well as apoptosis induction (20, 154). Its methylated analog PRIMA-1^Met^ (APR-246) is currently being studied in a phase I/II study in combination with pembrolizumab in subjects with solid malignancies (NCT04383938) (54). However, compound P53R3**** blocks glioma proliferation in a p53-dependent manner with a higher specificity and over a broader concentration range than PRIMA-1 (157). *In vivo* in GB, RITA**** showed synergistic effects when combined with TMZ and an inhibition of cell growth and stemness, as well as apoptosis induction. Interestingly, RITA acted independently of the p53 status (156). Protein expression studies showed that RITA suppressed cell proliferation by targeting the p53 associated protein ASK1 (156). Johansson et al. tested its efficacy in combination with the proteasome inhibitor bortezomib and despite showing specific single-agent activity in p53 *mut* cells, it did not strengthen bortezomib activity (154).

Since the p53 protein binds to DNA through a zinc-stabilized structurally complex domain, zinc plays a critical role in function of p53. It was shown that zinc aids in the transition of p53 mut into a functional conformation. In GB cells expressing the R273H mutation, this recovered their chemosensitivity (201). Also, NSC319726**** was able to restore the p53(R175) mutant to a functional p53 *wt* structure by acting as a zinc ionophore. This compound arrests GB-patient-derived cells, mediated by its binding to copper (155). Restoration of p53 function was also shown in glioma cells *in vitro* and *in vivo* upon exposure to a peptide called ****p53p-Ant (COOH-terminal peptide of p53 linked to the truncated homeobox domain of Antennapedia). The Fas extrinsic apoptotic pathway seemed to play a role in cell death induced by this protein (158).

Another possible approach to induce p53 reactivation is targeted gene therapy. This strategy enhanced radiosensitivity of p53 *wt* human glioma cells (202). The introduction of p53 *mut* into p53 *wt* human glioma cells promotes adenoviral-mediated p53 *wt* (****175H) gene transfer induced apoptosis (160). ****SGT-53 is a liposomal nanocomplex that delivers the p53 wt gene to tumor cells and has shown chemo-sensitization effects of GB *in vitro* and *in vivo* (28). However, the phase II trial of SGT-53 combined with TMZ in recurrent GB was terminated (NCT02340156) (54). The intratumoral administration of the adenovirus p53 gene was further explored in a phase I trial in patients with malignant primary glioma. However, a beneficial anti-tumor effect but widespread distribution of this agent remains a significant goal (159, 203). Nutlin-3 drug treatment combined with p19Arf gene**** transduction further activated p53 compared to single therapy in C6 GB cells. This vector is able to introduce p19Arf into p53 *wt* glioma cells, inducing viral expression of p19Arf with a subsequent activation of p53 (180). The adenovirus AdDelta24-p53****, which encodes the p53 protein and only replicates in Rb mutant cells, achieved potent anti-glioma effects *in vitro* when combined with RT (161). As an alternative for gene therapy, trans-membrane peptide therapy showed promising results in glioma cells. This technique uses a peptide derived from the MDM2 binding site of p53 (162).

#### Influencing the MDM2-Proteasome Interaction

Next to a direct MDM2-p53 interaction regulating the stability and ubiquitylation of p53, MDM2 also links with multiple subunits of the 26S proteasome increasing proteasomal turnover of p53. This lead to an increased interest in targeting the MDM2-26S proteasomal subunit interactions (106). This is achieved by SM JNJ-26854165 (Serdemetan),**** which binds the RING domain of MDM2. Results showed activity against both p53 *wt* and p53 *mut* GB cell lines and xenografts. However, a phase I clinical trial in advanced or refractory tumors did not proceed to phase II (204).

#### Inhibition of the E3 Ubiquitin Ligase Activity of MDM2

Multiple inhibitors of ubiquitin E3 ligases and deubiquitinating enzymes (DUBs) have been found to have potential anti-cancer properties. As reviewed by Antao et al., ‘thus far, USP2a, USP4, USP5, USP7, USP9X, USP10, USP11, USP15, USP24, USP29, and USP49 have been linked with p53 regulation’ (205). *In vitro* in glioma, the binding of USP2a**** to MDMX increased the mitochondrial location of p53 and stimulated apoptosis (164).

#### Natural Compounds

A handful of natural compounds/nutraceuticals have been studied for their MDM2 inhibitory or p53 activating effects in GB, as reviewed by Qin et al. (165). The BBB permeable nutraceutical *curcumin* **** has shown to exert anti-proliferative effects on glioma cells by modulating TP53/MDM2/MDMX/p14ARF signaling. In particular, curcumin upregulates p53 expression in GB *in vitro* and induces cell cycle arrest in a p53-dependent manner (167, 206). Pre-clinically in GB, chalcone derivatives**** and flavopiridol**** have shown to decrease MDM2 protein level or inhibit MDM2 expression at mRNA level, respectively (168, 207). ****Resveratrol showed inhibitory effects on the growth and metastatic capacity of both GB and GSCs, by partially acting through AKT inhibition and p53 activation, and suppressed GB growth *in vivo* (144).

### MDM2/MDMX Dual Inhibitors

For optimal efficacy, concomitant targeting of both MDM2 and MDMX may be necessary, since overexpression of MDMX can act as a MDM2 substitute, causing drug resistance (41, 208, 209). MDM2/X dual inhibitors have been reviewed elsewhere (37, 103). In a study by Chen et al., NSC623731**** was identified as the most potent dual specificity inhibitor *via* virtual screening and computational models and demonstrated anti-proliferative activity on the U87MG p53 *wt* GB cell line (171). In combined treatment strategies dual MDM2/X inhibitor RS3594**** and CXCRX inhibition presented synergic effects against GB pre-clinically (178). Other MDMX/2 inhibitors which have, to the best of our knowledge, not been studied for GB include SJ-172550, XI-006, XI-011, RO-2443, RO-5963, WK23 and WK298 (36, 37, 210, 211). RO-2443 and its chemically optimized analog RO-5963 are indolyl hydantoins which appeared to be MDM2/X antagonists with promising preclinical results (36, 210). In adult patients with advanced or metastatic solid tumors and in pediatric cancer, a phase I trial evaluating the MDM2/X inhibitor ****ALRN-6924 is currently active (NCT03725436, NCT03654716) (54).

Peptides and peptidomimetics in the p53/MDM2/MDMX circuitry are also emerging as interesting anti-cancer compounds given their increased selectivity linked to less toxicity and a lower propensity in developing cancer resistance, when compared to SMs (103). Liu et al. tested the D-peptide inhibitors of the p53-MDM2 interaction *^D^PMI-α* **** and *^D^PMI-#xD835;#xDEFD;* **** on U87MG and U251 GB cell lines and results confirmed p53 targeting. Interestingly, this group showed that D-peptide antagonists of MDM2 exert anti-GB effects *in vivo*, when encapsulated in liposomes linked to an integrin-targeting cyclic-RGD (Arg-Gly-Asp) peptide (212). Subsequently, a series of d-amino acid mutational PMI analogues, PMI-1-4****, were reported to have a higher proteolytic resistance and showed increased anti-tumor effects *in vitro*. Liposome-PMI-1 showed a stronger inhibitory activity against the U87MG p53 *wt* cell lines than Nutlin-3, without an effect on the U251 p53 *mut* GB cell line (169). PM2 potentiated the effect of protease inhibitor bortezomib in multiple GB cell lines by effectively inducing cell death after treatment. Interestingly, PM2 also radiosensitized p53 *wt* tumors but this needs to be confirmed in GB (137). ATSP-704****, a progenitor of the first stapled α-helical peptide entering clinical trials, binds both MDM2 and MDMX with high affinities and effectively activates the p53 pathway in tumors *in vitro* and *in vivo* but was not studied in GB. However, *in vivo*, [^3^H]-ATSP-7041 did not distribute to the brain and CNS tissues (213). Chen et al. tried to circumvent the BBB penetration issue by developing a cyclic RGD peptide-conjugated poly (ethylene glycol)-co-poly(lactic acid) polymeric micelle (RGD-M) that carried a stapled peptide antagonist of both MDM2 and MDMX (sPMI). ****RGD-M/sPMI inhibited GB growth both *in vitro* and *in vivo* (214).

## MDM2/X Inhibition and Other Combined Treatment Strategies

Although MDM2 inhibition has shown promising anti-cancer effects, not all p53 *wt* cell lines are sensitive to this treatment strategy and induction of apoptosis in p53 *wt* cell lines is sometimes limited (27, 189). In addition, therapeutic effects have been documented to be short-term due to acquired resistance or acquisition of p53 mutations (38, 107, 215). Hence, a combined treatment strategy might be necessary to reach optimal therapeutic effectiveness. Kocik et al., recently reviewed the current status of drug combinations to support MDM2 antagonists. These include targeted therapy, DNA damaging agents (chemical or IR) and apoptosis inducers. Targeted therapy strategies included tyrosine kinase inhibitors, Ras/Raf/MEK/MAPK inhibitors, cyclin-dependent kinase (CDK) inhibitors and PI3K/AKT/mTOR inhibitors. Dual inhibitors that have been reported to co-inhibit MDM2 include proteasome, histone deacetylases (HDAC), ATPase, XIAP, zinc, antibiotics, NF-κB pathway, translocator protein (TSPO), heat shock protein (HSP) inhibitors, integrin and mitotic inhibitors. Apoptotic inducers included BCL-2 inhibitors and tumor necrosis factor-related apoptosis-inducing ligand (TRAIL) agonists (38, 175, 176, 216–218).

Saiki et al. screened an 1169-compound library for potential compounds that synergize with MDM2 inhibition in inducing tumor cell death with the goal to circumvent resistance. They observed a robust synergy in inducing apoptosis with MEK or PI3K inhibitors, BH3 mimetics, BCR-ABL antagonists, and HDAC inhibitors (219). A phase II study combining MDM2 inhibitors in combination with immunotherapy, such as pembrolizumab (targeting programmed cell death protein 1) are currently undertaken in patients with advanced solid tumors, where p53 mutation status is an inclusion criteria (NCT03611868) (54, 220). Promising MDM2 inhibitor combination strategies for the treatment of GB will be briefly summarized in this section.

### MDM2-Chemotherapy

The synergism of combining MDM2 inhibition with chemotherapeutics has shown to be effective in AML and multiple trials are running in diverse tumor types (NCT04190550, NCT03725436, NCT03031730, NCT04113616, NCT04275518) (54). In GB, multiple pre-clinical studies have already proven that MDM2 inhibition induces chemosensitization, including Nutlin-3a, RG7112, spiropyrazoline oxindole 1a, RITA, SP-141 and SGT-53**** therapy (146, 149, 159, 160, 174, 217, 221). Nutlin-3a enhanced antitumor activity of TMZ in a humanized intracranial patient-derived xenograft model of GB (222). Nutlin-3a-loaded targeted micelles**** in combination with doxorubicin or the RGD MDM2/X targeting peptide-conjugated micelle (RGD-M/sPMI****) in combination with TMZ showed effective synergism against GB *in vitro* and *in vivo* (170, 172). Resveratrol**** also enhanced the sensitivity of TMZ resistant GB-initiating cells *via* the activation of the DSB/ATM/ataxia telangiectasia and Rad-3 related (ATR)/p53 pathway. However, blocking NF-κB-MGMT pathway thereby averting TMZ-resistance also plays a role (223). Genetic inhibition of MDM2 expression of glioma cells *in vitro* and *in vivo* by siRNA**** technologies or chemical inhibition by ****RG7112 also increased TMZ sensitivity of glioma cells, reversing the YB-1 protein mediated TMZ drug resistance (174).

### MDM2-Integrins

Merlino et al. investigated the effectiveness of peptidomimetic compounds targeting MDM2/X as well as α5β1/αvβ3 integrins. Studies were conducted on p53 *wt* glioma cells and showed that compound 9****** was the most effective in inducing long term cell cycle and proliferation arrest of cancer cells. Results also revealed a consequent reduction in cell invasion and migration, thereby confirming its potential as a novel class of integrin/MDM inhibitors (175).

### MDM2-AKT/mTOR

The interplay between the p53-MDM2 pathway and the PI3K/AKT pathway plays an important role in the determination of cell death and/or survival since this network involves two tumor suppressor genes (TP53 and PTEN) and two oncogenes (MDM2 and AKT) (224–226). AKT has shown to enhance MDM2 mediated p53 degradation (227). Data obtained from The Cancer Genome Atlas revealed that ∼88% of GB have activated PI3K pathways, which is linked with a poor prognosis (169, 228).

Among the different GB subtypes, the mesenchymal type shows the highest drug resistance, most frequent PTEN mutations (37%) and hyperactivation of PI3K/AKT (90). Daniele et al. explored the outcome of targeting both pathways by treating U87MG cells with the AKT/mTOR inhibitor *FC85* **** in combination with the established MDM2 inhibitor *ISA27* **** in an attempt to effectively treat GB by targeting their stem cells. Results showed a synergic effect on the inhibition of cell viability and on the reactivation of the p53 pathway leading to increased cell killing. Co-therapy also resulted in promoting differentiation, blocking proliferation and consequently apoptosis of GSCs (176). Synergy between MDM2 and PI3K/AKT/mTOR antagonists was also shown in liposarcoma and AML (226, 229). Interestingly, Saiki et al. noted that PI3K pathway mutations are not a prerequisite for this synergistic effect (219).

### MDM2-CDK4

Dual inhibitor *ent-4g* **** was developed to target both MDM2 and CDK4. Gene expression studies were performed on U251 GB cell lines and a noteworthy alteration in the cell cycle and p53 signaling pathways were observed. Flow cytometric results showed apoptotic induction and cell cycle arrest. This was confirmed in GB xenografts (230).

### MDM2-MEK

Pre-clinically, the MDM2 inhibitor RG7388 has shown promising results for the treatment of GB and synergism with irradiation but acquired resistance limits its potential. Combined treatment with the MEK inhibitor trametinib**** resulted in a restored sensitivity towards RG7388 therapy and a decrease in tumor growth *in vivo* (29).

### MDM2-CXCR4

Daniele et al. investigated the potential synergy between CXCR4 antagonists and MDM2/X inhibitors for GB therapy. The dual MDM2/X inhibitor RS3594 and the CXCR4 antagonist AMD3100**** presented synergic effects on cancer stem components and appears to be a valuable strategy to inhibit GB proliferation and reduce invasiveness (178).

### MDM2-V-ATPase

Inhibition of the proton pump V‐ATPase (vacuolar-type ATPase) by archazolid**** has shown to induce p53 protein levels in cancer cells. Subsequently, evidence was found that archazolid and nutlin‐3a combined therapy increased cell death in multiple p53 *wt* tumor cell lines and robustly activated IGFBP3 and Bax pro‐apoptotic pathways inducing caspase‐9 and PARP inactivation. Interestingly, the combination was more efficient in reducing U87MG GB growth *in vivo* compared to single dose treatment (179).

## Conclusion and Future Perspective

There is an urgency to develop novel agents directed at relevant pathways to increase effectiveness of GB therapy (231). Since 84% of GB patients show a deregulation of the p53-ARF-MDM2 pathway, the avenue of upregulating p53 and downregulating MDM2 has been explored extensively (4, 20). However, current data on single MDM2/X therapy in GB (see **Table 1**) is mostly preclinical and only a few clinical trials with MDM2 inhibitors are running in GB patients (NCT03107780, NCT03158389) (54, 154).

In addition, despite the acknowledged rationale, limited data is available on the use of MDM2/X inhibitors as radiosensitizers for the treatment of GB. p53 activation using MDM2/X inhibitors has shown radiosensitizing effects pre-clinically in lung cancer, prostate cancer, adenocarcinoma and colon cancer (21, 82, 108, 110, 112, 113). The first *in vitro* results on p53 *wt* GB cells show a potential synergy, but acquired resistance could be an issue (29, 142). This is further explored in GB patients under the active N²M² (NOA-20) trial, which investigates RT and molecularly matched targeted therapies, including RG7388 (idasanutlin) (NCT03158389) (NCT03158389) (54).

Importantly, dual inhibition of MDMX/MDM2 could help achieve full activation of p53, increasing therapeutic efficacy. In particular, inhibition of the p53-MDMX interaction presents an excellent opportunity for overcoming MDM2 inhibitor resistance when cancer cells overexpress MDMX (36). However, dual inhibitory drug development is proving to be challenging mainly due to the difference in the size of the Leu26 subpocket in MDM2 and MDMX (31). In addition, specific potent MDMX inhibitors are rare. There has been a recent trend in the emergence of peptides and peptidomimetics as attractive molecules due to their advantages compared to SMs, including their selectivity and tolerability, however, major drawbacks remain their intrinsic instability and their delivery to the target, including BBB crossing. Accordingly, only a few are currently in clinical trials compared to numerous SMs (103). The transfer of drug molecules to the tumor site could be improved using a wide range of carriers: liposomes, solid lipids nanoparticles, dendrimers, polymers, silicon or carbon materials and magnetic nanoparticles (232). As an example, ^D^PMI-α^16^, a D-peptide inhibitor of the p53–MDM2 interaction, encapsulated in liposomes decorated *via* a poly(ethylene glycol) spacer with a cyclic RGD peptide was effective in GB models (97, 103). Convection-enhanced delivery is also an option to improve delivery of targeted drugs to GB, applying local drug delivery that bypasses the BBB, while limiting associated systemic toxicities (233).

In light of recent RT developments and the promising role of particle therapy in GB treatment, more research is also needed to discover variations between different radiation qualities in inducing apoptosis signaling mechanisms, dependency of the p53 and MDM2 status and ROS production (120, 121). It is still not clear what determinants render cells susceptible towards cell death in response to MDM2 inhibitors, aside from functional p53 (39). More research will also help to clarify the determination of cell fate by the MDM2-p53 axis after DNA damage and other pathways in which the MDM2 protein and its diverse isoforms are involved. In cancer drug design, the p53 independent function of MDM2 in NBS1 regulation should be considered (39, 74, 234). For more open questions on the function of MDM2, see the recent publication of Dobbelstein et al. (39). In this regard, p53 targeted drugs including MDM2 inhibitors could elucidate new information.

Challenges such as acquired resistance and toxicity upon MDM2/X inhibition are not overcome yet, including effects on healthy tissues (29). New ways to interfere with MDM2 function are currently being developed, including proteolysis targeting chimera (PROTAC) degraders. However, it remains unclear whether these will improve efficacy without substantially increasing toxicity in human cancer patients (31, 39). Acquired resistance could be overcome by targeting multiple pathways concomitantly due to pathway redundancy, known to be present in GB. The first multi-targeted therapy strategies are only starting for GB and the ideal combination of inhibitors is unknown. Others drugs that might be worth to explore include MDM2 inhibitors with potent DNA damage repair pathway inhibitors targeting e.g. PARP, ATM, ATR, Checkpoint kinases CHK1, CHK2, WEE1, DNA-dependent protein kinase (DNA-PK) or other cell cycle pathway inhibitors targeting e.g Aurora kinase A and B, Polo-like kinase 1, RAD51 (4, 235, 236).

The main factor to select patients that are likely to benefit from MDM2/X treatment is the p53 status of the tumor and the level of MDM2 expression, although a combination of gene signatures might be necessary (27). For example, the CDKN2A gene encoding for tumor suppressor ARF that blocks MDM2 (76). MDM2 overexpression with or without gene amplification(s) is observed mainly in GB without p53 gene mutations (237). Up until now, the prognostic significance of MDM2 expression in GB is not confirmed (238). However, recent phase 1 clinical trials with SM MDM2 antagonists have indicated significant association between pre-treatment MDM2 expression levels and therapeutic response in patients with AML (25). Hence, there is a need for non-invasive predictive biomarkers for MDM2 targeted therapies. Fluorescence *in situ* hybridization and immunohistochemistry, the most commonly used methods for assessing MDM2 gene amplification and MDM2 protein overexpression in tumors, respectively, are invasive and do not permit monitoring the treatment response *in vivo* (239).

To address these needs, positron emission tomography (PET) and single-photon emission computed tomography (SPECT) radiotracers are promising to foresee a non-invasive way of imaging not only MDM2 but also other DNA damage repair proteins. This would lead to a more personalized approach, including treatment follow-up after MDM2/X therapy. PET/SPECT imaging agents for the oncoprotein MDM2 and p53 are limited at the moment. MDM2 antisense oligonucleotides were radiolabeled with [^99m^Tc], MDM2 inhibitor SP-141 was radiolabeled with [^18^F] and the peptide PM2 was radiolabeled with [^125^I], all in a pre-clinical stage (137, 240). Next to diagnostic information that radiolabeled MDM2/X inhibitors can reveal, they could also be useful for targeted radionuclide therapy when labelled with therapeutic radionuclides. In this way it would be possible to combine MDM2/X targeted treatment with targeted IR, taking advantage of the possible radiosensitizing effect of the combined treatment (241). However, this is a field that needs further investigation and more preclinical research.

## Author Contributions

JB: conception, literature search, write-up, revising, and approval. XM: conception, literature search, write-up, and approval. CV: conception, interpretation, revising, and approval. AH: interpretation, revising, and approval. All authors contributed to the article and approved the submitted version.

## Funding

This research was funded by the International Atomic Energy Agency (IAEA CRP E35010 (22248)).

## Conflict of Interest

The authors declare that the research was conducted in the absence of any commercial or financial relationships that could be construed as a potential conflict of interest.
